# An Ultra-Low-Voltage Transconductance Stable and Enhanced OTA for ECG Signal Processing

**DOI:** 10.3390/mi15091108

**Published:** 2024-08-30

**Authors:** Yue Yin, Xinbing Zhang, Ziting Feng, Haobo Qi, Haodong Lu, Jiayu He, Chaoqi Jin, Yihao Luo

**Affiliations:** School of Microelectronics, Northwestern Polytechnical University, 1 Dongxiang Road, Chang’an District, Xi’an 710129, China; yinyue@nwpu.edu.cn (Y.Y.); xinbing_zhang@mail.nwpu.edu.cn (X.Z.); ztfeng@mail.nwpu.edu.cn (Z.F.); haodonglu@mail.nwpu.edu.cn (H.L.); hejiayv@mail.nwpu.edu.cn (J.H.); jinchaoqi@mail.nwpu.edu.cn (C.J.); luoyihao@mail.nwpu.edu.cn (Y.L.)

**Keywords:** OTA, ultra-low-voltage, bulk-driven, subthreshold region, rail-to-rail, transconductance stabilization

## Abstract

In this paper, a rail-to-rail transconductance stable and enhanced ultra-low-voltage operational transconductance amplifier (OTA) is proposed for electrocardiogram (ECG) signal processing. The variation regularity of the bulk transconductance of pMOS and nMOS transistors and the cancellation mechanism of two types of transconductance variations are revealed. On this basis, a transconductance stabilization and enhancement technique is proposed. By using the “current-reused and transconductance-boosted complementary bulk-driven pseudo-differential pairs” structure, the bulk-driven pseudo-differential pair during the input common-mode range (ICMR) is stabilized and enhanced. The proposed OTA based on this technology is simulated using the TSMC 0.18 μm process in a Cadence environment. The proposed OTA consumes a power below 30 nW at a 0.4 V voltage supply with a DC gain of 54.9 dB and a gain-bandwidth product (GBW) of 14.4 kHz under a 15 pF capacitance load. The OTA has a high small signal figure-of-merit (FoM) of 7410 and excellent common-mode voltage (*VCM*) stability, with a transconductance variation of about 1.35%. Based on a current-scaling version of the proposed OTA, an OTA-C low-pass filter (LPF) for ECG signal processing with *VCM* stability is built and simulated. With a −3 dB bandwidth of 250 Hz and a power consumption of 20.23 nW, the filter achieves a FoM of 3.41 × 10^−13^, demonstrating good performance.

## 1. Introduction

In recent years, research in wearable technology and biomedicine have been continuously deepening and show a lot of potential [1,2,3,4]. In the integrated circuits of these fields, batteries are widely used, which leads to the contradiction between lightweight design and working time. As a result, the requirements for power supply and energy consumption are strict. Therefore, low voltage and low power design have become one of the hottest topics in analog integrated circuit design.

The amplifier is the most widely used and important module in analog circuits because it determines the performance of the entire circuit, such as filters, analog-to-digital converters (ADCs), digital-to-analog converters (DACs), and other circuits. For applications where the power supply voltage is lower than 1 V, especially below the MOS threshold voltage, circuits require amplifiers with high performance as well as the lowest possible supply voltage and power consumption. In low-voltage environments, amplifiers are required to have excellent input and output voltage ranges because the signals may be close to the power supply rail. As a solution to these needs, rail-to-rail amplifiers are frequently utilized in low voltage circuits, such as the band pass filter (BPF) or the low pass filter (LPF) used in an electrocardiograph (ECG) system and an electromyography (EMG) system [5,6,7], the comparator for ultra-low power successive approximation register analog-to-digital converter (SAR ADC) [8,9], detection circuits in biomedical implants [10], comparators for energy harvesting systems [11], and wireless sensors for internet of things (IoT) applications [12].

Several technologies were explored to realize amplifiers with rail-to-rail capabilities for ultra-low voltage environments. In [13], the authors use the complementary gate-driven structure to implement a rail-to-rail operational transconductance amplifier (OTA). However, the transconductance (*Gm*) is not stabilized when the common-mode voltage (*VCM*) of the input signals changes in the input common-mode range (ICMR). A rail-to-rail OTA with a voltage of 0.8 V is constructed in [14] using a floating-gate structure. In [15], a quasi-floating-gate structure is used to build an inverter that can be applied to the filter at 1.2 V. The floating-gate/quasi-floating-gate structure can achieve a rail-to-rail input common-mode range without complementary MOS pairs. However, the floating-gate/quasi-floating-gate structure must scale the input voltage with the capacitors, resulting in smaller transconductance and additional area consumption. Ref. [16] realizes a rail-to-rail op-amp at 1 V by stabilizing the operating voltage of the input transistors with level shifters. However, the shifters may cause deterioration in linearity, frequency response and power consumption.

Considering the limitations of the above technologies, bulk-driven technology has become a subject of great interest due to its inherent large common-mode input range and the capacity for independent bulk voltage control of transistors. There are two main problems with bulk-driven MOS: lower transconductance value than gate-driven and instability of transconductance with common-mode voltage. Many studies were conducted on the first problem. Three-stage high-gain OTAs are built-in [17,18] by using push–pull and pseudo-differential structures as the input stages, respectively. In [19], the flip voltage follower (FVF) structure is applied to build the OTA and current multiplexing technology is used to enhance gain. Refs. [20,21] use pseudo-differential pairs to construct a two-stage cascade amplifier and apply local positive feedback to enhance the gain. In addition, current multiplexing technology is used in [21] to boost the gain further. Ref. [22] uses a unique impedance enhancement technique to achieve higher gain. In [23], self-cascading transistors were used to construct a three-stage OTA with higher impedance and gain. Despite extensive work, there is still room to make improvements in relation to the first question. Compared to the first question, the second question is more significant and challenging. This is because the changing transconductance value directly impacts the amplifier’s performance, which could cause instability, additional nonlinearity, and other problems. However, it is frequently neglected with few relevant studies. Ref. [24] proposes a $G_{m}$-stabilizing technique that utilizes a variable positive feedback structure to adjust the working state of the current mirror and ensure the stability of $G_{m}$. Nonetheless, the $G_{m}$ still varies by 25%. In conclusion, the first problem requires further improvement, and there is an urgent requirement for a solution to the second problem.

In this work, a $G_{m}$-optimization technique is introduced to reduce $G_{m}$ variation in the ICMR of bulk-driven pseudo-differential pair input MOS pairs and enhance the equivalent transconductance. In Section 2, the $G_{m}$ instability of the bulk-driven MOS pairs is analyzed, and the $G_{m}$-optimization technique based on the current-reused complementary bulk-driven pseudo-differential pairs is proposed. In Section 3, an OTA with the proposed technique and an LPF using the proposed OTA are constructed and simulated.

## 2. The Transconductance-Optimization Technology

In this section, the $G_{m}$ variation in bulk-driven pseudo-differential (BDPD) pairs is analyzed, then the counteraction principle is introduced, and the current-reused complementary bulk-driven pseudo-differential pairs are proposed as the $G_{m}$-optimization technology.

### 2.1. Unstable Transconductance of BDPD in the Subthreshold Region

**Figure 1:**
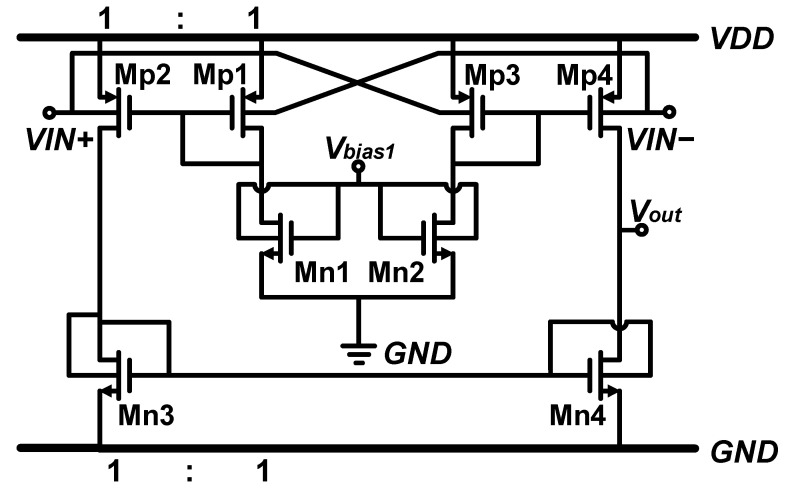
Conventional bulk-driven pseudo-differential input amplifier.

As Figure 1 shows, the pseudo-differential pair is more suitable for ultra-low-voltage environments due to its less voltage headroom and larger slew rate (SR) than the differential pair. In Figure 1, the nMOS transistors are made in the deep-N-well (DNW). And the current mirror transistors are biased by both the gate and bulk with a lower voltage, which is suitable for low-voltage supply.

In the bulk-driven pseudo-differential pair, the input signal is mapped to $V_{\mathrm{th}}$ to affect the pMOS transistor current by the body effect, which can be described as

(1) $$V_{\mathrm{th}}=V_{\mathrm{th}0}+\gamma (\sqrt{\left|2\Phi_{F}+V_{\mathrm{SB}}\right|}-\sqrt{\left|2\Phi_{F}\right|})$$

where $\Phi_{F}$ is the Fermi potential, γ is the body effect factor, $V_{\mathrm{BS}}$ is the body-source voltage and $V_{\mathrm{th}0}$ is the threshold voltage when $V_{\mathrm{BS}}$ = 0. In the pMOS transistor, the γ and $\Phi_{F}$ are both negative.

The MOS transistors are often biased in the subthreshold region in ultra-low-voltage circuits. The current of a pMOS transistor in the subthreshold region is expressed as

(2) $$I_{\mathrm{DS}}=I_{S}\left(\frac{W}{L}\right)\exp\left(q\frac{V_{\mathrm{SG}}+V_{\mathrm{th}}}{\mathrm{nkT}}\right)\left[1-\exp\left(-q\frac{V_{\mathrm{SD}}}{\mathrm{kT}}\right)\right]$$

where $I_{S}$ is the characteristic current, T is the absolute temperature, n is the inclination of the curve in weak inversion, k is the Boltzmann constant, and q is the charge of the electron or hole. This expression is a consensus among the EKV [25], BSIM3v3 [26] and ACM [27] models. The transistor will be saturated in the subthreshold region when $V_{\mathrm{SD}}>3\mathrm{kT}/q$. When the pMOS transistor is controlled only by the gate, the threshold voltage $V_{\mathrm{th}}$ and $V_{S}$ are both fixed. The gate-transconductance $g_{m}$ is defined as

(3) $$g_{m}=-\frac{\partial I_{\mathrm{DS}}}{\partial V_{G}}=\frac{\partial I_{\mathrm{DS}}}{\partial {(V}_{\mathrm{SG}}+V_{\mathrm{th}})}=q\frac{I_{\mathrm{DS}}}{\mathrm{nkT}}>0$$

In this case, the bulk transconductance $g_{\mathrm{mb}}$ can be written as

(4) $$g_{\mathrm{mb}}=-\frac{\partial I_{\mathrm{DS}}}{\partial V_{B}}=-\frac{\partial I_{\mathrm{DS}}}{\partial {(V}_{\mathrm{SG}}+V_{\mathrm{th}})}\times \frac{\partial {(V}_{\mathrm{SG}}+V_{\mathrm{th}})}{\partial V_{B}}=g_{m}\times \frac{-\partial V_{\mathrm{th}}}{\partial V_{B}}=g_{m}\times \frac{-\gamma }{2\sqrt{\left|2\Phi_{F}+V_{\mathrm{SB}}\right|}}$$

Obviously, $g_{\mathrm{mb}}$ is equal to the product of gate-transconductance $g_{m}$ and the coefficient $-\partial V_{\mathrm{th}}/\partial V_{B}$. For the bulk-driven pseudo-differential pair, the change of $V_{B}$ is equal to the change of VCM. As a result, $g_{\mathrm{mb}}$ is directly affected by the different levels of VCM.

### 2.2. Counteraction of Transconductance Variation in nMOS and pMOS BDPD Pairs

The versions of Equation (4) for pMOS and nMOS can be written, respectively, as

(5) $$g_{\mathrm{mb},p}=g_{m,p}\times \frac{\partial V_{\mathrm{th},p}}{\partial V_{B,p}}=g_{m,p}\times \frac{{-\gamma }_{p}}{2\sqrt{-2\Phi_{F,p}+\left.V_{\mathrm{BS},p}\right.}}=q\frac{I_{\mathrm{DS},p}}{n_{p}\mathrm{kT}}\times \frac{{-\gamma }_{p}}{2\sqrt{-2\Phi_{F,p}+\left.\mathrm{VCM}-\mathrm{VDD}\right.}}$$

(6) $$g_{\mathrm{mb},n}=g_{m,n}\times \frac{\partial V_{\mathrm{th},n}}{\partial V_{B,n}}=g_{m,n}\times \frac{\gamma_{n}}{2\sqrt{2\Phi_{F,n}-V_{\mathrm{BS},n}}}=q\frac{I_{\mathrm{DS},n}}{n_{n}\mathrm{kT}}\times \frac{\gamma_{n}}{2\sqrt{2\Phi_{F,n}-\left.\mathrm{VCM}\right.}}$$

Obviously, $g_{\mathrm{mb},p}$ and $g_{\mathrm{mb},n}$ show opposite trends in response to VCM. Therefore, the change of transconductances can be offset partly after summing the $g_{\mathrm{mb}}$ of pMOS and nMOS. Considering the change of $g_{\mathrm{mb}}$ as linear, the variation of $g_{\mathrm{mb},p}$ and $g_{\mathrm{mb},n}$ can be written as

(7) $$∆g_{\mathrm{mb},p}=q\frac{I_{\mathrm{DS}}}{n_{p}\mathrm{kT}}\left(\frac{{-\gamma }_{p}}{2\sqrt{-2\Phi_{F,p}}}-\frac{{-\gamma }_{p}}{2\sqrt{-2\Phi_{F,p}-V\mathrm{DD}}}\right)$$

(8) $$∆g_{\mathrm{mb},n}=q\frac{I_{\mathrm{DS}}}{n_{n}\mathrm{kT}}\left(\frac{\gamma_{n}}{2\sqrt{2\Phi_{F,n}-V\mathrm{DD}}}-\frac{\gamma_{n}}{2\sqrt{2\Phi_{F,n}}}\right)$$

And the relative variation of $g_{\mathrm{mb},p}$ and $g_{m,n}$ can be defined as the ratio of $∆g_{\mathrm{mb}}$ to $g_{\mathrm{mb},\max}$, written as

(9) $${∆}^{\prime }g_{\mathrm{mb},p}=\left|\frac{{-\gamma }_{p}}{2\sqrt{-2\Phi_{F,p}}}-\frac{{-\gamma }_{p}}{2\sqrt{-2\Phi_{F,p}-V\mathrm{DD}}}\right|/\frac{{-\gamma }_{p}}{2\sqrt{-2\Phi_{F,p}}}=\frac{\sqrt{-2\Phi_{F,p}}-\sqrt{-2\Phi_{F,p}-V\mathrm{DD}}}{\sqrt{-2\Phi_{F,p}-V\mathrm{DD}}}$$

and

(10) $${∆}^{\prime }g_{\mathrm{mb},n}=\frac{\sqrt{2\Phi_{F,n}}-\sqrt{2\Phi_{F,n}-V\mathrm{DD}}}{\sqrt{2\Phi_{F,n}}}$$

Generally, $∆g_{\mathrm{mb},p}$ is not equal to $∆g_{\mathrm{mb},n}$ when the static current of nMOS and pMOS is the same. To best counteract $g_{\mathrm{mb}}$ variation, the bias current ratio of nMOS and pMOS, denoted as R, should be

(11) $$\;R=R_{0}=\frac{g_{m,p}}{g_{m,n}}\frac{\left(\frac{{-\gamma }_{p}}{2\sqrt{-2\Phi_{F,p}}}-\frac{{-\gamma }_{p}}{2\sqrt{-2\Phi_{F,p}-V\mathrm{DD}}}\right)}{\left(\frac{\gamma_{n}}{2\sqrt{2\Phi_{F,n}+V\mathrm{DD}}}-\frac{\gamma_{n}}{2\sqrt{2\Phi_{F,n}}}\right)}$$

The derivatives of $g_{\mathrm{mb},p}$ and $g_{\mathrm{mb},n}$ are, respectively, monotonically increasing and monotonically decreasing, written as

(12) $$\frac{dg_{\mathrm{mb},p}}{d\mathrm{VCM}}=q\frac{I_{\mathrm{DS},p}}{n_{p}\mathrm{kT}}\times \frac{\gamma_{p}}{4{\left(-2\Phi_{F,p}+\left.\mathrm{VCM}-\mathrm{VDD}\right.\right)}^{\frac{3}{2}}}<0$$

and

(13) $$\frac{dg_{\mathrm{mb},n}}{d\mathrm{VCM}}=q\frac{I_{\mathrm{DS},n}}{n_{n}\mathrm{kT}}\times \frac{-\gamma_{n}}{4{\left(2\Phi_{F,n}-\left.\mathrm{VCM}\right.\right)}^{\frac{3}{2}}}>0$$

And $∆g_{\mathrm{mb},p}$ and $∆g_{\mathrm{mb},n}$ are equal to the integral of their derivatives from 0 to VDD. For the total transconductance $g_{\mathrm{mb},\mathrm{total}}$ = $g_{\mathrm{mb},p}$ + $g_{\mathrm{mb},n}$, the derivative of $g_{\mathrm{mb},\mathrm{total}}$ is

(14) $$\frac{dg_{\mathrm{mb},\mathrm{total}}}{d\mathrm{VCM}}=\frac{dg_{\mathrm{mb},p}}{d\mathrm{VCM}}+\frac{dg_{\mathrm{mb},n}}{d\mathrm{VCM}}=q\frac{I_{\mathrm{DS},p}}{n_{p}\mathrm{kT}}\times \frac{\gamma_{p}}{4{\left(-2\Phi_{F,p}+\left.\mathrm{VCM}-\mathrm{VDD}\right.\right)}^{\frac{3}{2}}}+q\frac{I_{\mathrm{DS},n}}{n_{n}\mathrm{kT}}\times \frac{-\gamma_{n}}{4{\left(2\Phi_{F,n}-\left.\mathrm{VCM}\right.\right)}^{\frac{3}{2}}}$$

assuming that $\frac{dg_{\mathrm{mb},\mathrm{total}}}{d\mathrm{VCM}}$ = 0 when $\left.\mathrm{VCM}\right.\;$=${\left.\left.\mathrm{VCM}\right.\right.}_{0}$. Then, ∆gmb,total is equal to the integral of $\frac{dg_{\mathrm{mb},\mathrm{total}}}{d\mathrm{VCM}}$ from 0 to ${\left.\left.\mathrm{VCM}\right.\right.}_{0}$. According to the law of integration, it is obvious that $\left|∆g\mathrm{mb},\mathrm{total}\right|\;$<$\;\left|∆g\mathrm{mb},p\right|$ and $\left|∆g\mathrm{mb},\mathrm{total}\right|$ <$\;\left|∆g\mathrm{mb},n\right|$.

If gmb,p and gmb,n are completely linear, then ∆gmb,total = 0. However, even if gmb,p and gmb,n are not completely linear, $\left|∆gmb,\mathrm{total}\right|$ is quite a smaller value than $\left|∆gmb,p\right|$ or $\left|∆gmb,n\right|$.

In summary, the transconductance variation can be reduced from $∆g_{\mathrm{mb},p}$ for the pMOS type to $∆g_{\mathrm{mb},\min}$ for the complementary type.

Previous studies have shown that $\Phi_{F,p}$ (negative) and $\Phi_{F,n}$ (positive) have small approximate linear changes towards the zero potential when the temperature increases. For simplicity, only $\Phi_{F,p}$ and $\Phi_{F,n}$ are considered as the functions of temperature in the coefficient $\partial V_{\mathrm{th}}/\partial V_{B}$. Formulas (9) and (10) indicate that ${∆}^{\prime }g_{\mathrm{mb},p}$ and ${∆}^{\prime }g_{\mathrm{mb},n}$ are relative to $\Phi_{F,p}$ and $\Phi_{F,n}$, and they show the same trend in response to changes in temperature. In another way, ${∆}^{\prime }g_{\mathrm{mb},p}$ and ${∆}^{\prime }g_{\mathrm{mb},n}$ show the same trend in response to changes in temperature. Therefore, the temperature has little effect on $R_{0}$.

However, the temperature has an obvious effect on the absolute value of bulk transconductance $g_{\mathrm{mb}}$. Formulas (5) and (6) indicate that $\partial V_{\mathrm{th},p}/\partial V_{B,p}$ and $\partial V_{\mathrm{th},n}/\partial V_{B,n}$ are relative to $\Phi_{F,p}$ and $\Phi_{F,n}$, and they both increase slightly as the temperature goes up. While the gate-transconductance $g_{m}$ decrease as the temperature goes up. Overall, the absolute value of bulk transconductance decreases as temperature goes up. And it can be solved by a current source with a temperature coefficient.

### 2.3. Current-Reused Bulk-Driven Pseudo-Differential Pairs

**Figure 2:**
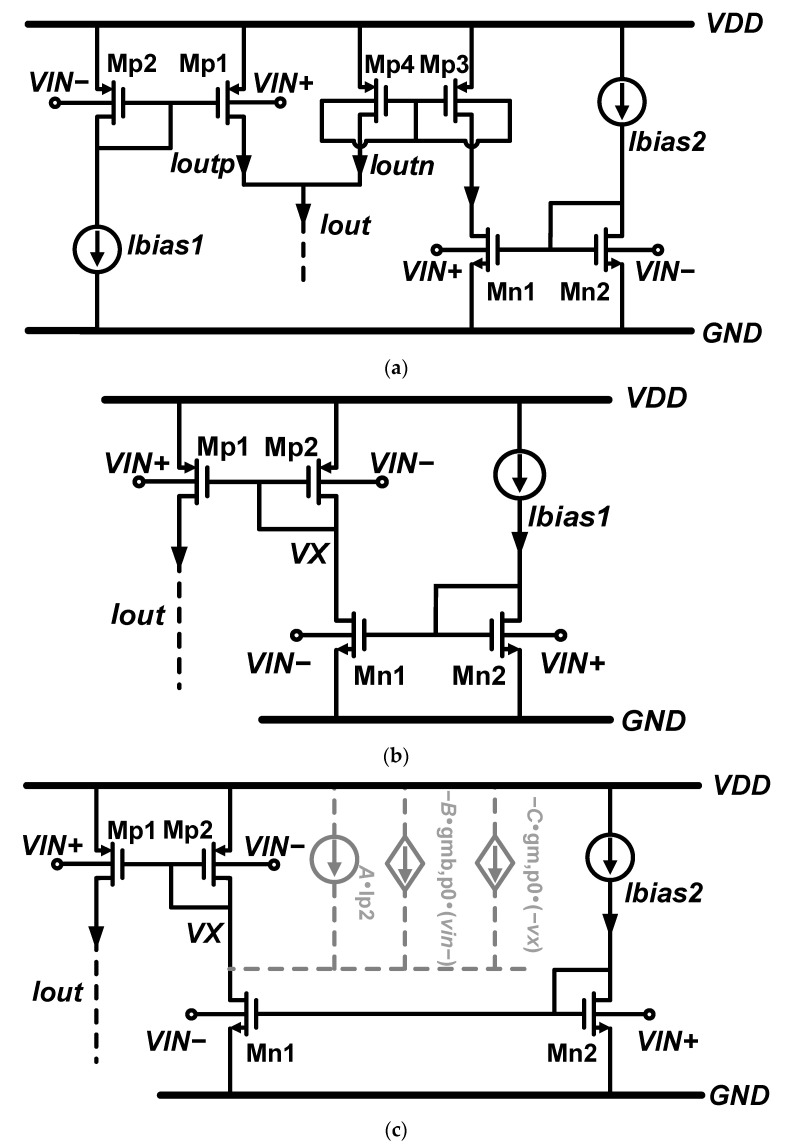
(**a**) Complementary bulk-driven pseudo-differential pairs, (**b**) current-reused bulk-driven pseudo-differential pairs, (**c**) current-reused bulk-driven pseudo-differential pairs with correction.

Figure 2a shows the complementary bulk-driven pseudo-differential pairs circuit. The circuit could add $g_{\mathrm{mb},n}$ and $g_{\mathrm{mb},p}$ and achieve $R=R_{0}$ by using a current mirror and independent current sources.

However, replicating the small signal current of the pMOS pair by the current mirror structure results in additional current and lower current utilization efficiency compared to a single MOS pair.

The “current-reused bulk-driven complementary pseudo-differential pairs” are adopted to improve the efficiency of current utilization. As shown in Figure 2b, an nMOS bulk-driven pseudo-differential pair replaces the original current source of the pMOS pair. Setting the size and current ratio between Mp1 and Mp2 is 1:1, and the total equivalent transconductance $G_{m}$ can be written as

(15) $$G_{m}=g_{\mathrm{mb},p0}+g_{\mathrm{mb},n0}$$

where $g_{\mathrm{mb},p0}=g_{\mathrm{mb},p1}=g_{\mathrm{mb},p2}$, $g_{\mathrm{mb},n0}=g_{\mathrm{mb},n1}=g_{\mathrm{mb},n2}$.

Unfortunately, the bias current match of the nMOS and pMOS in Figure 2b cannot be achieved. To achieve the best current matching, a negative impedance branch can be introduced next to Mp2. The branch provides more bias current for the nMOS pair and increases the pMOS pair’s small signal current replication ratio. Hence, the ratio of nMOS and pMOS equivalent currents noted as R, is corrected to be greater than 1. At the same time, this branch can be used as a small signal input of pMOS. In summary, this branch can perform current correction and further improve the overall transconductance. As Figure 2c shows, A, B and C represent the branch’s direct current, small signal transconductance and impedance.

Setting the current of Mp1 the same as Figure 2b, the total transconductance provided by this structure is

(16) $$G_{m}=g_{\mathrm{mb},p0}\left(\frac{2+B-C}{2-2C}\right)+g_{\mathrm{mb},n0}\left(\frac{1+A}{1-C}\right)$$

Then, R is equal to $\left(2+2A\right)/\left(2+B-C\right)$. Obviously, R = $R_{0}$ can be achieved by controlling A, B, and C. The negative conductance is important because it contributes to $G_{m}$-boost by increasing the total transconductance.

## 3. The Proposed Transconductance Stable and Enhanced OTA and the ECG LPF

In this section, an OTA with stable and enhanced transconductance is constructed based on the proposed current-reused pseudo-differential pairs, and an LPF using the proposed OTA is built. The working principle and performance of the OTA are analyzed, and the simulation results of the OTA and LPF are presented.

### 3.1. Design and Performance of the Proposed OTA and LPF

**Figure 3:**
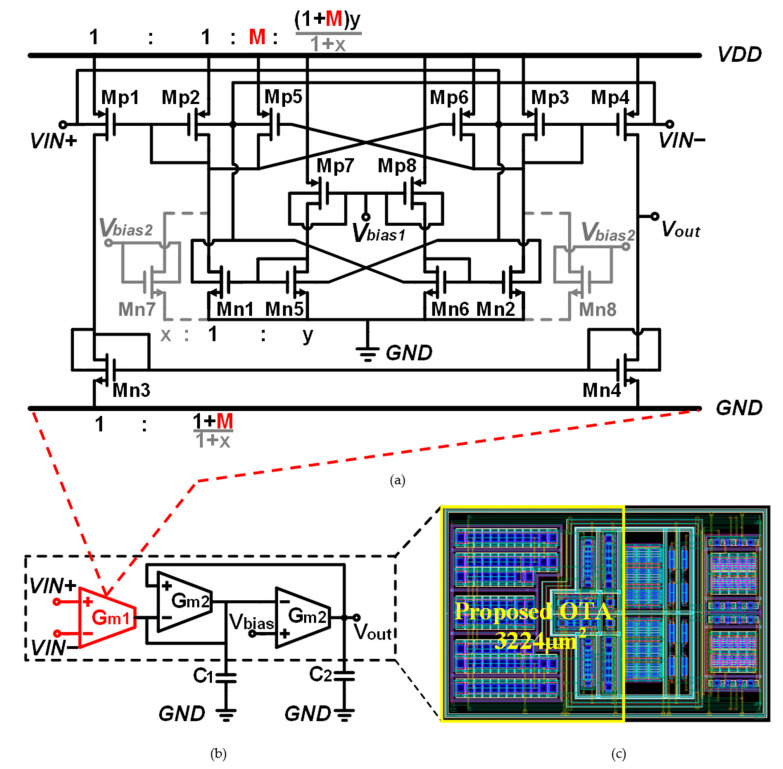
(**a**) The proposed OTA, (**b**) the LPF, and (**c**) the layout of BPF without capacitances.

Figure 3a shows the circuit of the proposed OTA. To achieve the additional current injection mentioned in Section 2, input transistors Mp5 and Mp6 are added, and their gates are connected to cross-coupled mode. And, the size ratios between the same type of transistors are marked by the proportion formulas in the circuit. For example, the size ratio of Mp5 to Mp2 is 1:M. Similar to the conventional OTA shown in Figure 1, all the nMOS transistors are made in the deep-N-well (DNW).

The transistors’ size ratios of the same type determine their current ratios, as well as the transconductance ratios. For the current mirror in bulk-driven pseudo-differential pairs, it can be proved that the synchronous change of the master MOS transistor’s size and the driving current does not affect the working state of the slave MOS transistor and the size of the total bulk transconductance of the pseudo-differential pair. Therefore, the current ratio of Mn5 to Mn1 can be set to y:1 to reduce the current consumption. Assuming that the total static current of the Mp2 and the Mp5 branches is $I_{0}$, and $g_{\mathrm{mb}}$ corresponding to $I_{0}$ is $g_{\mathrm{mb},n0}$ and $g_{\mathrm{mb},p0}$ and ignoring the channel length modulation effect, the total transconductance can be written as

(17) $$G_{m}=\frac{2}{1-M}\times \left(\frac{1}{1+M}\times g_{\mathrm{mb},p0}+g_{\mathrm{mb},n0}\right)$$

Assuming that the bias current of the Mp1 transistor in the two OTAs is the same, the traditional OTA equivalent transconductance value can be written as

(18) $$G_{m}=2\times \left(\frac{1}{1+M}\right)\times g_{\mathrm{mb},p0}$$

The transconductance enhancement of the proposed OTA is reflected in 1−M and $g_{\mathrm{mb},n0}$.

The ratio R in the proposed OTA is equal to 1+M. A simulation of the circuit in Figure 2a shows that *R*0 is equal to 1.4. Considering that M is set to 0.7 for transconductance-boost, there are two schemes to correct R. The first scheme is to adjust M to 0.4 in the proposed OTA. However, in this case, $g_{\mathrm{mb},p}$ and $g_{\mathrm{mb},n}$ are both compressed by nearly half. The second scheme is to add Mn7 as an independent current source to reduce the weight of $g_{\mathrm{mb},n}$ individually. As Figure 3a shows, the current ratio between Mn7 and Mn1 is x:1; then, the corrected total transconductance can be written as

(19) $$G_{m}=\frac{2}{1-M}\times \left(\frac{1}{1+M}\times g_{\mathrm{mb},p0}+\frac{1}{1+x}\times g_{\mathrm{mb},n0}\right)$$

The ratio R is corrected to 1+M/1+x. It is obvious that R can be corrected to about $R_{0}$ by setting x to $(1+M)/R_{0}$. What is more, the current of additional M7 can be independently set to achieve the varying value of $R_{0}$ in real circuits.

The noise of the conventional OTA and the proposed OTA can be expressed as

(20) $$\;\bar{v_{n,A\mathrm{orB}}^{2}}=\;N\times \bar{v_{n,0}^{2}}$$

where $\bar{v_{n,0}^{2}}=\left[\frac{8\mathrm{kT}(g_{m,p1}+g_{m,n3})}{3g_{\mathrm{mb},p0}^{2}}+\frac{\left(K_{p}g_{m,p1}^{2}/W_{p1}L_{p1}+K_{n}g_{m,n3}^{2}/W_{n3}L_{n3}\right)}{g_{\mathrm{mb},p0}^{2}f{\;C}_{\mathrm{ox}}}\right]$, and the coefficient N in the noise formula can be written as

(21) $$\;N_{A}=\frac{1}{2}(1+M{)}^{2}$$

(22) $$\;N_{B}=\frac{1}{4}\frac{(1+M{)}^{2}(1-M{)}^{2}}{\;\left.{\left[1+\frac{g_{\mathrm{mb},n}}{g_{\mathrm{mb},p}}\frac{(1+M)}{(1+x)}\right]}^{2}\right.}\left\{\left.\left.1+\frac{(1+M\left.)\right.}{(1-M{)}^{2}}\left[1+\frac{1}{(1+x{)}^{2}y}\right]\right\}\right.\right.$$

Obviously, $N_{B}{\leq N}_{A}$ can be guaranteed using a y value bigger than a certain value $y_{0}$.

### 3.2. The ECG Filter with the Proposed OTA

The original ECG signal typically has frequencies within 250 Hz [28] with a voltage peak of 4 mV. For the ECG signal, an LPF and a low noise amplifier (LNA) are needed to suppress high-frequency noise and amplify the amplitude. An OTA-C filter with a certain gain is suitable for low-frequency ECG signal processing. However, the filter’s gain is determined by the $G_{m}$ of the input OTA, which is affected by the VCM of the input signal and requires much current. In this section, an LPF using the proposed OTA is built to overcome the mentioned shortages.

As Figure 3b shows, a second-order OTA-C LPF is built with the proposed OTA for ECG signal processing. The transmission function of the filter can be written as

(23) $$H(s)=\frac{\frac{G_{m1}G_{m3}}{C_{1}C_{2}}}{s^{2}+s\frac{G_{m2}}{C_{1}}+\frac{G_{m2}G_{m3}}{C_{1}C_{2}}}$$

Setting $G_{m2}=G_{m3}=G_{\mathrm{mL}}$, the $\omega_{0}$ and the quality factor Q can be expressed as

(24) $$\omega_{0}=\frac{G_{\mathrm{mL}}}{\sqrt{C_{1}C_{2}}}$$

and

(25) $$Q=\sqrt{\frac{C_{1}}{C_{2}}}$$

The passband gain of the filter is

(26) $$\mathrm{Av}p=\frac{G_{m1}}{G_{\mathrm{mL}}}$$

In this filter, *G_m_*_1_ is implemented by the proposed OTA with the modified bias current. Then *G_m_*_2_ and *G_m_*_3_ are implemented by the high-linearity pseudo-differential OTAs. The high-linearity OTA, in which the original passive degeneration resistor is replaced by MOS transistors with independent bias, is an improved version of the OTA proposed in [29]. The proposed OTA achieves a large transconductance with less current than the high-linearity OTA, which means lower noise than using high-linearity OTAs in direct parallel or multiplier current modes. In this case, *G_m_*_1_ contributes the most the noise of the filter. Moreover, the BPF’s performance is almost unaffected by the common-mode level of the differential input signal, which expands the limitations on the front measurement circuit.

The filter is set to Butterworth type (*Q* = 0.707) and has a low-pass cutoff frequency of 250 Hz by setting $C_{1}$ and $C_{2}$ to 7.11 pF and 14.22 pF. The cutoff frequency of the filter is determined by *G_m_*_2_ and *G_m_*_3_ and can be changed by the bias current of a high-linearity OTA. Similarly, the passband gain can be set by the bias current of the proposed OTA.

### 3.3. Simulation Results and Comparison

The OTA and the LPF are simulated with the TSMC 0.18 μm process. In the proposed OTA, M is set to 7/10, x to 1/5, and y to 3/5. The layout of LPF is shown in Figure 3c.

**Figure 4:**
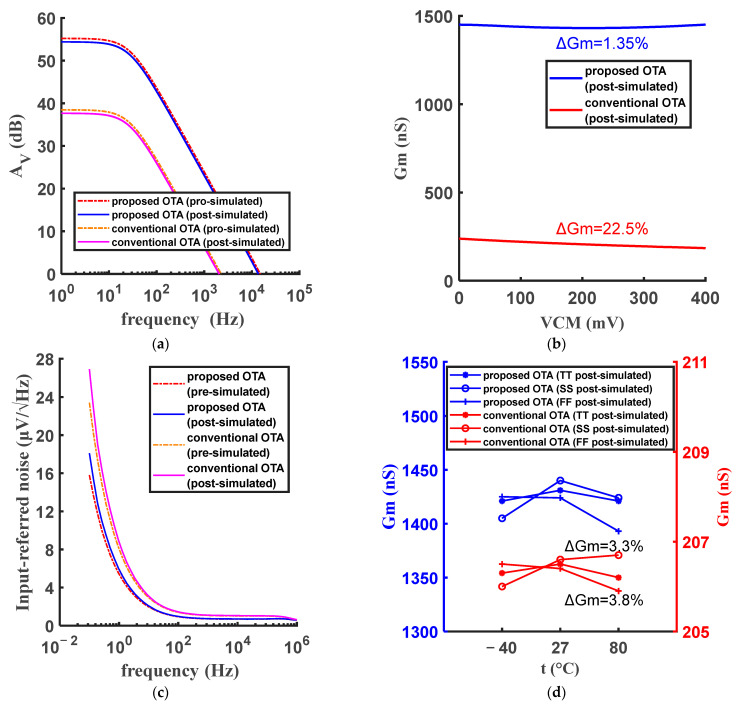
Pre-simulation and post-simulation results of the proposed and conventional OTAs: (**a**) amplitude-frequency characteristic, (**b**) Gm at different VCM, (**c**) input-referred noise @ VCM = 200 mV and (**d**) Gm at different process corners and temperatures.

Figure 4 shows the performance comparison of the proposed amplifier and the conventional amplifier. As Figure 4a shows, the DC gain of the proposed OTA increases by about 16.7 dB compared with conventional OTA. As Figure 4b shows, the variation of *G*_m_ is reduced from 22.5% in the conventional OTA to 1.35% in the proposed OTA. The input-reference noises (IRN) of the two OTAs @ VCM = 200 mV are shown in Figure 4c, indicating that the proposed OTA has a better noise performance. Figure 4d shows the $G_{m}$ of the two OTAs with a particular temperature coefficient current source at different process corners and temperatures. Under the TT process corner, both high and low temperatures would affect the operation of the current mirrors. And temperature would further affect the replication of current and $G_{m}$. The FF and SS process corner conditions could compensate the effect of low and high temperatures, respectively. Therefore, $G_{m}$ exhibits the characteristics shown in Figure 4d. The variation of $G_{m}$ of the proposed OTA at different process corners and temperatures is 3.3%, indicating that the proposed OTA can be process-robust and temperature-robust.

**Figure 5:**
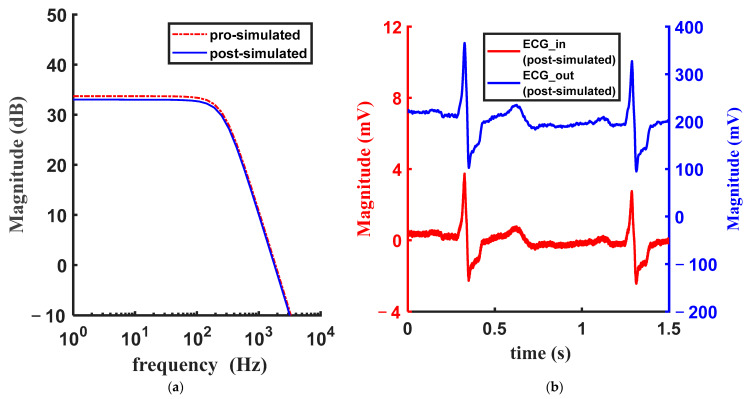
Pre-simulation and post-simulation results of the filter: (**a**) amplitude-frequency characteristic of the LPF and (**b**) the input and output ECG signals.

Figure 5 shows the performance of the two amplifiers and the LPF. Figure 5a shows the amplitude-frequency characteristic of the LPF. Considering a peak amplitude of the ECG signal of 4 mV and the supply voltage of the circuit of 0.4 V, a passband gain of 33 dB is appropriate. The ECG noisy signal applied to the input of the LPF containing 0.2 mV @ 2 kHz noise and the filtered output signal is shown in Figure 5b.

As Table 1 shows, the proposed OTA has a relatively small supply voltage of 0.4 V and a power consumption of 29.15 nW. It also shows two large FoM values with small signal performance. These parameters indicate that the proposed OTA has a high transconductance efficiency. When VCM changes, the proposed OTA has a minimum transconductance change degree of 1.35% and achieves the largest FoM value with VCM stability. Considering the supply voltage, the figure-of-merit with the OTA’s VCM robustness is defined as

(27) $${\mathrm{FoM}}_{S,3}=\frac{\mathrm{GBW}\;[k\mathrm{Hz}]\times \mathrm{CL}\;\left[\mathrm{pF}\right]}{I_{\mathrm{total}}\;[\mu A]\times \mathrm{GBW}\_\mathrm{variation}\times 100}$$

Table 2 shows the performance comparison of the proposed LPF with other low-voltage biomedical filters. The proposed LPF has a minimum supply voltage, low power consumption, and low noise. The dynamic range (DR), as the ratio of input rms value @ 1% total harmonic distortion (THD) to the input-referred noise of the LPF, is greater than 40 dB, although it is limited by the passband gain.

The following figure-of-merit [38], which is very widespread, was used to establish an objective comparison

(28) $$\mathrm{FoM}=\frac{\mathrm{Power}\;\left[W\right]}{N\times f_{c}\;[\mathrm{Hz}]\times \mathrm{DR}\;\left[\mathrm{abs}.\right]}$$

where N and $f_{c}$ represent the number of poles and bandwidth (or the center frequency in the bandpass filter) of the filter.

## 4. Conclusions

In this paper, the variation of the bulk transconductance in pMOS and nMOS body-driven pseudo-differential pairs was investigated, and the current reused bulk-driven complementary pseudo-differential pairs are constructed, which combines the two types of transconductance efficiently. A bulk transconductance optimization technique based on current reused bulk-driven complementary pseudo-differential pairs is proposed. The technique can stabilize the bulk transconductance of MOS pairs and improve transconductance efficiency. An ultra-low-voltage bulk-driven OTA is constructed using this technique. Compared with the previous OTAs, the proposed OTA has excellent transconductance efficiency and minimal transconductance variation with VCM. Finally, the proposed OTA is used to build an OTA-C LPF for ECG signal processing with the same VCM stability and low power consumption. The ECG filter possesses relatively excellent performance. The proposed OTA with low power consumption is also suitable for electromyogram (EMG), electroencephalogram (EEG) and other signal processing in the biomedical field.

## Figures and Tables

**Table 1:** Performance comparison of sub-1 V OTAs.

Parameter	This Work ^(a)^ 2024	[30] ^(a)^ 2021	[31] ^(a)^ 2020	[32] ^(a)^ 2019	[33] ^(a)^ 2019	[34] ^(a)^ 2018
Technology [nm]	180	180	180	180	180	180
Operation mode	BD	BD	BDandGD	BD	BD	BD
Supply [V]	0.4	0.5	±0.3	0.6	0.3	±0.5
Power [nW]	29.15	45.5	252	180	12.6	62,000
DC gain [dB]	54.4	83.7	53.81	75.39	68.9	61.7
GBW [kHz]	13.7	8.7	56.62	74.27	2.94	1510
CL [pF]	15	15	30	20	30	35
IRN [nV/√Hz]	700@ 10 kHz	650@ 1 kHz	265@ 5 kHz	209@ 40 kHz	1600	69@ 1 MHz
Phase margin [°]	73.2	59	73.8	78.9	54.7	83.2
FoM_S,1_* [kHz•pF/μA]	**2820**	1434	4044	4951	2100	852
FoM_S,2_** [kHz•pF/μW]	**7050**	2868	6740	8252	7000	852
Gm(GBW) variation	**1.35%**	/	>5%	>9.5%	>40%	41.2%
FoM_S,3_ [kHz•pF/μA]	**2089**	/	<809	<522	<53	20.7

^(a)^ post-simulated results. * ${\mathrm{FoM}}_{S,1}=\mathrm{GBW}\;[Hz]\times \mathrm{CL}\;\left[\mathrm{pF}\right]/I_{\mathrm{total}}\;[\mu A]$. ** ${\mathrm{FoM}}_{S,2}=\mathrm{GBW}\left[\mathrm{Hz}\right]\times \mathrm{CL}\;\left[\mathrm{pF}\right]/\mathrm{Power}\;[\mu W].$

**Table 2:** Performance comparison of sub-1 V biomedical filters.

Parameter	This Work ^(a)^ 2024	[7] ^(a)^ 2022	[35] 2020	[36] ^(a)^ 2020	[37] 2019
Technology [nm]	180	180	180	180	180
Structure	BD-OTA-C	BD-DDA-C	SF-C	BD-QFG-OTA-C	GD-OTA-C
Order	2	2	4	2	5
Supply [V]	0.4	0.5	0.5	1	1
Power [nW]	20.5	62.6	3.69	1100	41
Gain [dB]	33	39.6	−5.6	39.9	−7
Bandwidth [Hz]	250	0.1–150	200	0.266–2.8k	250
IRN [μVrms]	19.9	59	91.9	3.15	134
DR [dB]	41.5	/	48.5	/	61.2
FOM [J]	3.45 × 10^−13^	/	0.17 × 10^−13^	/	5.36 × 10^−13^

^(a)^ post-simulated results.

## Data Availability

Data are contained within the article; further inquiries can be directed to the corresponding author.
